# Complete Genome Sequence of *Bacillus megaterium* Siphophage Stahl

**DOI:** 10.1128/genomeA.00857-15

**Published:** 2015-08-06

**Authors:** Ashley M. Brizendine, Simon Rousseau, Adriana C. Hernandez, Gabriel F. Kuty Everett

**Affiliations:** Center for Phage Technology, Texas A&M University, College Station, Texas, USA

## Abstract

Stahl is a siphophage active against *Bacillus megaterium*, a Gram-positive bacterium often used as a model system in research and as a protein production strain in industrial applications. Here, we present the complete annotated genome of phage Stahl and describe its major features.

## GENOME ANNOUNCEMENT

*Bacillus megaterium* is an important bacterium to research and industry. Due to its large size, it is extensively utilized in molecular biology studies. It is nonpathogenic and contains no endotoxins, making it ideal for large-scale protein synthesis (1). Bacteriophages capable of infecting *B. megaterium* can further the research and industrial applications of this bacterium with their various uses in genetic manipulation. Here, we describe phage Stahl, a siphophage isolated against an asporogenic strain of *B. megaterium*, Km Sp^−^(ATCC 13632).

Stahl was isolated from a soil sample collected in College Station, TX. Phage DNA was sequenced in an Illumina MiSeq 250-bp paired-end run with a 550-bp insert library at the Genomic Sequencing and Analysis Facility at the University of Texas (Austin, TX). Quality-controlled trimmed reads were assembled to a single contig at 67.9-fold coverage using Velvet version 1.2.10. The contig was confirmed to be complete by PCR using primers that face the upstream and downstream ends of the phage DNA. Products from the PCR amplification of the junctions of concatemeric molecules were sequenced by Sanger sequencing (Eton Bioscience, San Diego, CA). Genes were predicted using GeneMarkS (2) and corrected using software tools available on the Center for Phage Technology (CPT) Galaxy instance (https://cpt.tamu.edu/galaxy-public/) (3, 4). Morphology was determined using transmission electron microscopy performed at the Texas A&M University Microscopy and Imaging Center.

Stahl has a double-stranded DNA (dsDNA) genome that is 80,148 bp in length, has a G+C content of 35.27%, and has a coding density of 89.9%. Based on sequence comparison using Emboss Stretcher, Stahl shares 85.6% and 84% nucleotide sequence identity to *B. megaterium* siphophages Slash (GenBank accession no. NC_022774) and Staley (GenBank accession no. NC_022767), respectively (2).

Few replication/recombination proteins were identified (Holliday junction resolvase, helicase, primase, and an exonuclease). Stahl encodes proteins for transcriptional regulation including two sigma factors and a transcription factor. Although the tape measure and tail fiber structural genes were identified, other structural proteins were not identified likely due to sequence divergence and lack of homology to other phages. A class II holin (two transmembrane domains in an N-in, C-in topology) was annotated along with two peptidoglycan-degrading enzymes: a secreted amidase and a soluble peptidase (5).

Stahl encodes genes that are suggestive of a temperate lifestyle, such as a Rha superfamily protein (IPR014054), which contains a bacteriophage P1 Ant1-like C-terminal domain (IPR005039) and an integrase/recombinase (IPR004107, IPR013762). Rha, a protein encoded by a variety of temperate phages, prevents growth on bacterial strains that lack an integration host factor (6); Ant1 is required to maintain lysogeny in bacteriophage P1 (7). The integrase is a putative tyrosine recombinase that facilitates integration of the phage genome into that of its host (8).

Additionally, Stahl encodes a secreted LysM (IPR018392, peptidoglycan-binding domain) and CAP (IPR014044, cysteine-rich secretory, antigen 5, and pathogenesis-related) domain-containing protein. This protein has been identified in few phages, and its role in the phage infection cycle is unknown.

### Nucleotide sequence accession number.

The genome sequence of phage Stahl was contributed to GenBank under the accession no. KP696447.
